# Development of a career questionnaire for medical undergraduates using Mokken scale analysis

**DOI:** 10.1186/s12909-022-03340-8

**Published:** 2022-04-15

**Authors:** Yizhuo Gao, Xue Bai, Le Sun, Dong Jia

**Affiliations:** 1 Department of Pulmonary and Critical Care Medicine, Shengjing Hospital of China Medical University, No. 36, Sanhao Street, Shenyang, China; 2 Department of Health Management, Shengjing Hospital of China Medical University, No. 36, Sanhao Street, Shenyang, China; 3 Department of Graduate Medical Training, Shengjing Hospital of China Medical University, No. 36, Sanhao Street, Shenyang, China; 4 Department of Pediatrics, Shengjing Hospital of China Medical University, No. 36, Sanhao Street, Shenyang, China; 5 Department of Emergency Medicine, Shengjing Hospital of China Medical University, No. 36, Sanhao Street, Shenyang, China

**Keywords:** Career choice, Mokken scale analysis, Questionnaire, Undergraduate, Medical School

## Abstract

**Background:**

Individual-centred career questionnaires are important for understanding the motivations of medical students. This study aimed to collect validity evidence of a questionnaire to measure the career choice of medical undergraduates.

**Methods:**

A cross-sectional survey was sent to third-year undergraduate students at a Chinese university-affiliated hospital. The questionnaire was formed using items that were selected after a systematic literature review. Item reduction was conducted using Mokken scale analysis, followed by reliability and validity testing, which described the validity evidence of the content, response process and internal structure.

**Results:**

The preliminary 20-item questionnaire was returned by 213 undergraduate students (response rate: 86.59%). To construct a monotone homogeneity model, 6 items were removed after testing for unidimensionality, local independence, and latent monotonicity according to the sequence. The final questionnaire included 14 items in two subscales: a 10-item ‘career advantage’ subscale and a 4-item ‘career disadvantage’ subscale. The questionnaire was judged to be acceptably reliable (Molenaar-Sijtsma method: 0.87 and 0.75, Cronbach’s alpha: 0.87 and 0.74) and to have good construct validity (χ2/df: 1.748, normed fit index: > 0.9, comparative fit index: > 0.9, root mean square error of approximation: 0.05–0.08). Male and female undergraduates had different responses regarding their salary, subspecialty, career prospects, and ability to serve their relatives. Male undergraduates might be more willing to accept on-call positions and have subspecialties with greater likelihoods of patient–physician conflict.

**Conclusion:**

We used Mokken scale analysis to develop and collect evidence of the validity of a 14-item questionnaire regarding career preferences among Chinese medical undergraduate students. This short and simple questionnaire may provide a suitable tool for exploring insights regarding the motivations of Chinese medical students.

**Supplementary Information:**

The online version contains supplementary material available at 10.1186/s12909-022-03340-8.

## Background

Unbalanced medical industrial structure and unequal distributions of health care resources have always been a global problem affecting most countries [1, 2], and have attracted more public attention during the SARS-CoV-2 outbreak [3, 4]. In China, institution-level differences in salaries, career prospects and occupational stress vary widely, which may lead to these conditions [2, 5]. For example, an urban physician in a tertiary hospital may receive a relatively higher salary and have better career prospects but might have an increased workload and higher likelihood of patient–physician conflict [6, 7]. In addition, there are broad gaps among employment in different subspecialties [8]. For example, a relative shortage of paediatricians was serious at each hospital level [9, 10]. Despite the active implementation of hierarchical care, partnership assistance and specialist training, better results have still not been achieved [11]. A more effective medical reform strategy should be based on not only the status quo of medical work but also undergraduates’ actual demands. The Chinese Medical Doctor Association devotes much effort conducting surveys on the practice of physicians. In 2009, 2011, 2014 and 2017, four surveys on the practice of physicians were conducted. In 2018, the Chinese Medical Doctor Association announced a survey of over 140 thousand doctors in approximately 44,600 hospitals across the country, showing the current status of Chinese doctors' practice in detail [11]. Despite being an important implementer of medical work, reports on the status quo of talent are rare [5].

Some previous studies have assessed the motivations of Chinese medical university students from different perspectives; however, these studies revealed quite unilateral or inconsistent findings [12, 13]. One possible reason for these different conclusions may be the absence of a reliable, standardized, and uniform questionnaire for evaluating the motivations of Chinese medical undergraduates. Furthermore, previous questionnaires have generally been based on expert opinions or the existing literature, and their utility has not been systematically described or evaluated, which affects the clear comparisons of the findings. Therefore, the present study aimed to develop and collect validity evidence of a questionnaire to measure the career choices of Chinese medical undergraduate students. This questionnaire may provide a more objective basis for evaluating medical students’ employment status during the economic changes from the SARS-CoV-2 pandemic.

## Methods

### Item selection and revision

This survey was performed as part of a career choice research program for undergraduate medical students at China Medical University. All students received a QR code link to the survey as part of their course work, although participation was voluntary and anonymous. All participants received verbal and online explanations of the questionnaire and were informed that informed consent would be assumed if they completed and submitted the questionnaire. The questionnaire was administered, and data were collected using a free online tool (https://www.wjx.cn).

Four educational and clinical experts (YZG, XB, LS, and DJ) guided and supervised the item selection process. The MEDLINE (PubMed) and EMBASE databases were searched using ‘career choice medical undergraduates [text word]’ and ‘career choice medical survey [text word]’ to identify potentially relevant reports that were published between 1 January 1990 and 30 August 2019. Two investigators (YZG and DJ) independently screened the results using the titles, abstracts, and full texts (where appropriate), and reports were included if the two investigators reached consensus regarding their relevance.

The questionnaire items included demographic items (age and sex) and career choice items. The career choice items were extracted from the relevant reports, categorized, and combined when different items had similar meanings. The items were then sorted in descending order according to their frequency of use and discussed by the group of experts.

A questionnaire was created after the items that were judged to be meaningful and suitable for Chinese medical students were selected and translated into Chinese for ease of use. Nineteen students completed a pilot survey between 20 August 2020 and 28 August 2020 for the evaluation of the preliminary questionnaire. All items were evaluated using a 7-point Likert scale, with responses scored from 1 (strongly disagree) to 7 (strongly agree) [14].

### Item analysis and reduction

A cross-sectional survey was performed between 01 September 2020 and 30 November 2020, with data extraction performed on 11 December 2020. Participants from eight third-year student classes who were studying clinical medicine at Shengjing Hospital were enrolled.

Mokken scale analysis is a type of non-parametric item response theory analysis that can reduce the number of questionnaire items based on assumptions of unidimensionality, local independence, and latent monotonicity [15, 16]. Mokken scale analysis was performed to form unidimensional scales of polytomous items and to explore the factor structure of each scale. First, an automated item selection procedure was performed via Mokken scale analysis to identify the unidimensional scales (item sets) from the item pool. The item structure was evaluated based on each item’s pattern and scalability (*Hi*) [17], and individual items that had *Hi* values of > 0.3 and paired items with scalability (*Hij*) of > 0 were selected [18]. Second, we tested each scale based on the assumption of local independence using two indices (W1 and W3) of conditional associations [19]. Third, monotonicity was evaluated using an item response function graph and the related indices (e.g., vi, zsig, and crit statistics) [20]. Fourth, invariant item ordering was performed based on the assumption of non-intersecting item response functions [21]. We also evaluated whether the monotone homogeneity model or the double monotonicity model fit the data better, with the results judged as insufficient (*H*^*T*^ < 0.3), weak (0.3 ≤ *H*^*T*^ < 0.4), moderate (0.4 ≤ *H*^*T*^ < 0.5), or strong (*H*^*T*^ ≥ 0.5) [16]. The number of items in the questionnaire was then sequentially reduced based on the results from the analyses described above.

### Reliability and factor structure

Mokken’s Rho was used to estimate the reliability of each subscale’s internal consistency. Reliability was assessed using the Molenaar-Sijtsma method [22], Cronbach’s alpha [23], Guttman’s method (lambda-2) [24], and the latent class reliability coefficient [25].

Factor structure validity was evaluated using confirmatory factor analysis to evaluate relationships between the questionnaire items and scales. The four indices were chi-squared/degrees of freedom (χ^2^/df), root mean square error of approximation (RMSEA), normed fit index (NFI), and comparative fit index (CFI) [26].

### Statistical analysis

The statistical analysis was performed using R software (version 4.0.2) [27] with the ‘mokken’ package [15], ‘lavaan’ package [28], and ‘semPlot’ package [29]. Continuous variables are expressed as the mean ± standard deviation.

## Results

### Item selection and revision

**Fig. 1:**
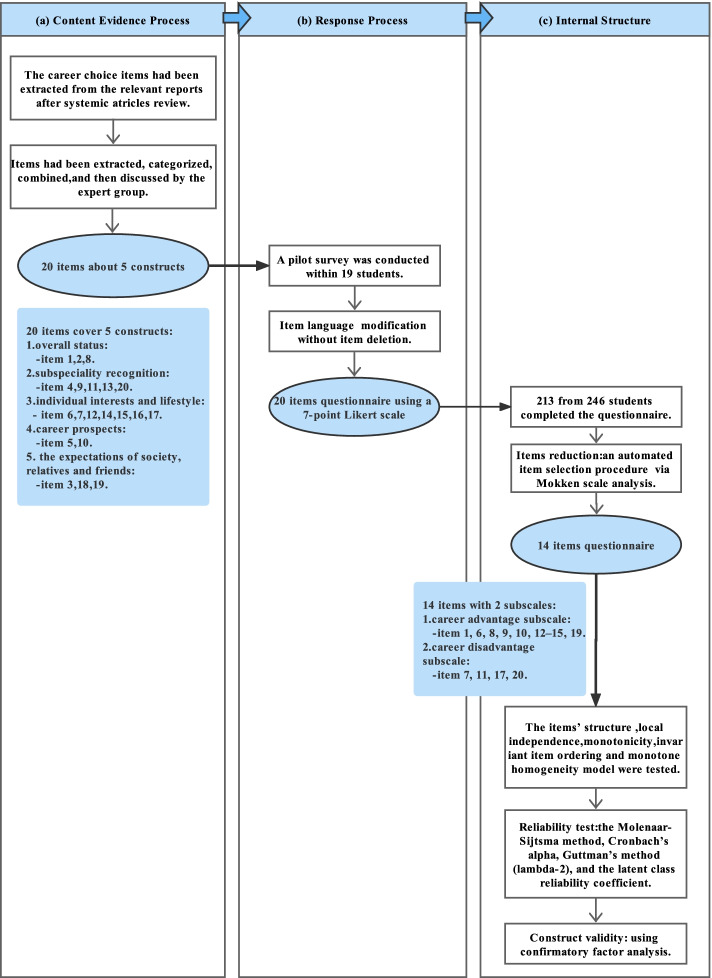
Questionnaire development flowchart. The flowchart shows the sequential process, including (**a**) the collection of the content evidence process, (**b**) the collection of evidence for the responses process, and (**c**) the examination of the tool's internal structure

**Fig. 2:**
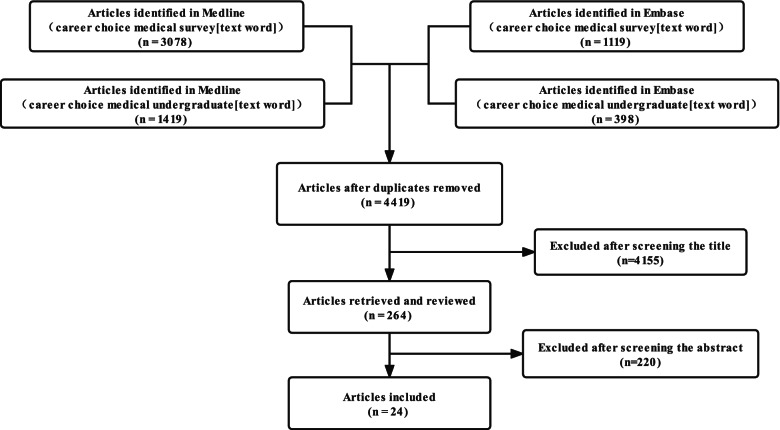
Literature search flowchart. The literature search strategy and the number of studies filtered out after each step

A flow chart summarizing the entire collection process for evidence of validity is shown in Fig. 1. The literature search process is shown in Fig. 2. After removing duplicate results, the titles and abstracts of 4,419 potentially relevant articles were screened, and 24 articles were ultimately included for item extraction. The extracted items were listed based on their frequency of use, and similar items were combined by the group of experts. This process identified 20 items for the questionnaire, and a pilot survey with 19 students (9 males and 10 females) was conducted. All 19 students returned the questionnaire feedback form, and more than 90% (18/19) of the students agreed that all the items should be kept and revised some expression phrases to different degrees. Based on this feedback, no item was deleted, and the language was modified in some instances for greater clarity. The English version questionnaire was shown in Supplementary Table 1. Table 1 shows the preliminary questionnaire items consisting of two aspects about hospitals (Items 1–7) and subspecialties [8–20]. The included items covered five constructs: overall status (Items 1, 2, and 8), subspeciality recognition (Items 4, 9, 11, 13, and 20), individual interests and lifestyle (Items 6, 7, 12, 14, 15, 16, and 17), career prospects (Items 5 and,10), and expectations of society, relatives and friends (Items 3, 18, and 19).

**Table 1:** Preliminary questionnaire items

Item	Scores	*Hi*	SE	Scale
Sex (male)	213 (104)			^a^
Age (years)	21.2 ± 0.77			^a^
1. I prefer to choose general and famous hospital(i.e. tertiary hospitals)	6.04 ± 0.91	0.371	0.05	1
2. I prefer to choose medium degree hospital(i.e. secondary hospitals)	4.39 ± 1.30			^a^
3. I prefer to meet the needs of society (i.e., community or private hospitals)	3.65 ± 1.38			^a^
4. I prefer to work at a specialized hospital with a good reputation	5.33 ± 1.27			^a^
5. I prefer to work at a hospital with room for promotion	4.97 ± 1.28			^a^
6. I prefer to work at a hospital near my hometown	5.89 ± 0.99	0.506	0.037	1
7. I am willing to work at a hospital with greater occupational stress	4.71 ± 1.27	0.481	0.056	2
8. I prefer a subspecialty that will provide a high salary	6.00 ± 0.83	0.357	0.054	1
9. I prefer a subspecialty with prestigious experts	5.84 ± 0.97	0.533	0.037	1
10. I prefer a subspecialty with good career prospects	6.11 ± 0.92	0.496	0.04	1
11. I prefer a more competitive subspecialty	4.34 ± 1.32	0.386	0.06	2
12. I prefer an interesting subspecialty	6.19 ± 0.84	0.535	0.041	1
13. I prefer a subspecialty with greater job satisfaction	6.08 ± 0.90	0.496	0.045	1
14. I prefer a subspecialty that fits my character and work style	6.13 ± 0.86	0.516	0.039	1
15. I prefer a subspecialty that will have a limited effect on my leisure time	5.64 ± 1.16	0.357	0.046	1
16. I prefer a subspecialty with few night or overtime shifts	5.23 ± 1.30			^a^
17. I am willing to choose a subspecialty where I will always be on-call	4.25 ± 1.62	0.529	0.044	2
18. I am willing to choose a subspecialty that is recommended by family or friends	3.89 ± 1.80			^a^
19. I am willing to choose a subspecialty where I can serve my relatives	5.79 ± 1.08	0.345	0.055	1
20. I am willing to choose a subspecialty with a greater likelihood of patient-physician conflict	2.46 ± 1.06	0.422	0.049	2

^a^ indicates items that were filtered out during the automated item selection procedure

### Participants and responses

A total of 246 undergraduate students were invited to participate, and 213 students (104 male students and 109 female students) completed all items in the questionnaire (response rate: 86.59%). The participants’ demographic characteristics (sex and age) and item scores are summarized in Table 1, Supplementary Table 2, and Supplementary Fig. 1. The scores for all 20 items ranged from 2.46 ± 1.06 to 6.19 ± 0.84, with a score of 4 indicating a neutral response. Thus, generally negative responses were observed for Item 3 (I prefer to meet the needs of society [i.e., community or private hospitals]), Item 18 (I am willing to choose a subspecialty that is recommended by my family or friends), and Item 20 (I am willing to choose a subspecialty with a greater likelihood of patient–physician conflict). Generally, neutral and positive responses were observed for the other items.

### Item analysis and reduction

The 20 items were subjected to an automated item selection procedure using Mokken scale analysis, which identified two unidimensional scales. Scale 1 was defined as the ‘career advantage’ subscale and included 10 items: Items 1, 6, 8, 9, 10, 12–15, and 19. Scale 2 was defined as the ‘career disadvantage’ subscale and included 4 items: Items 7, 11, 17, and 20. Items 2–5, 16, and 18 were filtered out because the *Hi* values were below the accepted cut-off of 0.3 (Table 1). A local independence evaluation did not exclude any of the items within each subscale, and no locally dependent item pairs were identified based on the W1 and W3 values. The monotonicity plot showed no significant monotonicity for any of the items within the subscales (values of zero for vi, zsig, and crit) (Supplementary Fig. 2). Invariant item ordering revealed limited accuracy of the item ordering on the ‘career advantage’ subscale (*H*^*T*^ = 0.107) but accurate ordering on the ‘career disadvantage’ subscale (*H*^*T*^ = 0.828). Thus, the questionnaire consisted of two subscales that fulfilled the monotone homogeneity model but did not fulfil the double monotonicity model.

### Reliability

Table 2 shows the reliability results based on the Molenaar-Sijtsma method, Cronbach’s alpha, Guttman’s method (lambda-2), and the latent class reliability coefficient. The questionnaire was considered acceptably reliable because all the estimates provided values > 0.7.

**Table 2:** Reliability estimates

	MS	Cronbach’s alpha	Lambda-2	LCRC
Career advantage	0.87	0.87	0.88	0.88
Career disadvantage	0.75	0.74	0.75	0.77

*MS* Molenaar-Sijtsma method, *LCRC* latent class reliability coefficient

### Factor structure

Confirmatory factor analysis was conducted, and the model was adjusted using the modification index (Table 3 and Supplementary Fig. 3). The modified model reflected an acceptable fit of the data, based on a χ^2^/df value of < 3, an NFI of > 0.9, a CIF of > 0.9, and an RMSEA value of 0.05–0.08.

**Table 3:** Confirmatory factor analysis indices before and after modification

	χ^2^	df	χ^2^/df	RMSEA	NFI	CFI
Model before modification	341.65	76	4.495	0.128	0.748	0.79
Model after modification	120.63	69	1.748	0.059	0.911	0.959

*RMSEA* root mean square error of approximation, *NFI* normed fit index, *CFI* comparative fit index

## Discussion

The present study used non-parametric Mokken scale analysis to collect evidence of the validity of a simple career questionnaire for Chinese undergraduate medical students. The questionnaire items were initially selected from previously published articles and then organized and combined to create a 20-item preliminary questionnaire. We then used Mokken scale analysis to create two subscales that fit the monotone homogeneity model, which included a 10-item ‘career advantage’ subscale and a 4-item ‘career disadvantage’ subscale. The final questionnaire exhibited acceptable reliability and construct validity. The questionnaire was developed for this study and has not previously been published elsewhere.

Relative to parametric item response theory models, non-parametric models have fewer data constraints. In the present study, Mokken scale analysis was used because it is flexible and relies less on item score distributions and sample sizes, which were important characteristics for the present study’s generally skewed item scores and limited sample size [30].

The final questionnaire consisted of two subscales regarding career choice advantages and disadvantages, which fit the sample data relatively well, as tested by confirmatory factor analysis. The mokken analysis of questionnaire reveals two subscales as same as the confirmatory factor analysis two-factor structure, including consistent items composition. This means that the questionnaires of medical students' career choice are stable because classical test theory and item response theory show the same solution.

Many of the items were similar to items that have been used for previous surveys in China and other countries [13, 31]. Thus, the final 14-item questionnaire appears to be more concise, reliable, and valid. When using our questionnaire, the researcher may refer invariant item ordering to reorder items according to their facility. The item ordering on the ‘career advantage’ subscale is 0.107, which means that the questions are ordered so we can formulate the questionnaire in a certain order (e.g., score order) for students to answer. However accurate ordering on the ‘career disadvantage’ subscale is 0.828 so their order seems to have little effect on the results, and there is no need to specifically consider the order of the 4 disadvantage items.

The validity evidence collection of this questionnaire was based on the data of one single centre in China. Compared with foreign questionnaires, it may be more representative of the actual local situation. If this questionnaire can be further promoted in the future, it might reflect information from other parts of China. This questionnaire might represent the career choices of local medical students, and the results might provide more information for employment education as well as effective data for curriculum development in our centre or wider areas.

### Limitations

The present study has several limitations. First, we only considered undergraduate students at a single Chinese centre, and more comprehensive results would be provided by surveying other regions or conducting a nationwide survey to validate and modify this questionnaire. Second, we only considered undergraduate students who had not started their clerkship, although some studies have indicated that clinical clerkships might affect students’ subspecialty choices. Therefore, we hope to prospectively evaluate how the survey responses change before and after the students have completed their clerkships. It may also be prudent to perform a large multicentre study to determine whether the questionnaire can be improved.

## Conclusion

We used Mokken scale analysis to collect validity evidence of a simple career questionnaire for Chinese medical undergraduate students. The questionnaire includes a 10-item ‘career advantages’ subscale and a 4-item ‘career disadvantages’ subscale. The development of this questionnaire might provide an effective tool for career intention surveys and curriculum development.

## Supplementary Information

**Additional file 1:** **Supplementary Table 1.** Preliminary questionnaire (English language version). This is the questionnaire sent to 246 students after the pilot survey, including an explanation section and items.

**Additional file 2:** **Supplementary Table 2.** Item score details. The table show the number of students per item corresponding to each score.

**Additional file 3:** **Supplementary Fig. 1.** Items score plot. Boxplots represent the fraction of different scores for each item.

**Additional file 4:** **Supplementary Fig. 2.** Graphic display of monotonicity. One graph for each item plots the estimated item response function.

**Additional file 5:** **Supplementary Fig. 3.** Model of cconfirmatory factor analysis. The Figure shows the relationship within each item and between two subscales.

## Data Availability

The data used in this study may be available on Harvard Dataverse: https://doi.org/10.7910/DVN/OISZTE.

## Abbreviations

χ2/df: Chi-squared divided by degrees of freedom
RMSEA: Root mean square error of approximation
NFI: Normed fit index
CFI: Comparative fit index
No.: Number
