# Supplementary material for: Development of a career questionnaire for medical undergraduates using Mokken scale analysis
Source: BMC Med Educ. 2022 Apr 15;22:286. doi: 10.1186/s12909-022-03340-8 (PMC9011374; doi:10.1186/s12909-022-03340-8)
Supplement: Supplementary file 2 — Additional file 2: Supplementary Table 2. Item score details. The table show the number of students per item corresponding to each score. [file 12909_2022_3340_MOESM2_ESM.docx]

Additional files 2.

Table 2 Item score details

**Score=1 Score=2 Score=3 Score=4 Score=5 Score=6 Score=7**

| Item 1 | 0 | 0 | 1 | 14 | 36 | 87 | 75 |
| --- | --- | --- | --- | --- | --- | --- | --- |
| Item 2 | 8 | 11 | 19 | 71 | 67 | 29 | 8 |
| Item 3 | 16 | 28 | 47 | 70 | 31 | 18 | 3 |
| Item 4 | 5 | 0 | 6 | 39 | 62 | 61 | 40 |
| Item 5 | 4 | 2 | 9 | 69 | 56 | 44 | 29 |
| Item 6 | 0 | 0 | 1 | 24 | 40 | 81 | 67 |
| Item 7 | 2 | 7 | 21 | 68 | 56 | 41 | 18 |
| Item 8 | 0 | 0 | 0 | 9 | 46 | 95 | 63 |
| Item 9 | 0 | 0 | 1 | 21 | 50 | 80 | 61 |
| Item 10 | 0 | 1 | 0 | 12 | 33 | 82 | 85 |
| Item 11 | 4 | 11 | 34 | 80 | 39 | 33 | 12 |
| Item 12 | 0 | 0 | 0 | 11 | 25 | 90 | 87 |
| Item 13 | 0 | 0 | 0 | 15 | 32 | 86 | 80 |
| Item 14 | 0 | 0 | 0 | 14 | 25 | 93 | 81 |
| Item 15 | 0 | 2 | 4 | 37 | 40 | 72 | 58 |
| Item 16 | 3 | 6 | 4 | 46 | 58 | 60 | 36 |
| Item 17 | 12 | 22 | 28 | 63 | 31 | 40 | 17 |
| Item 18 | 21 | 40 | 24 | 51 | 31 | 26 | 20 |
| Item 19 | 2 | 0 | 0 | 23 | 52 | 73 | 63 |
| Item 20 | 40 | 79 | 58 | 28 | 8 | 0 | 0 |

The table show the number of students per item corresponding to each score.

Item 1. I prefer to choose general and famous hospital （i.e., tertiary hospitals）

Item 2. I prefer to choose medium degree hospital （i.e., secondary hospitals）

Item 3. I prefer to meet the needs of society (i.e., community or private hospitals)

Item 4. I prefer to work at a specialized hospital with a good reputation

Item 5. I prefer to work at a hospital with room for promotion

Item 6. I prefer to work at a hospital near my hometown

Item 7. I am willing to work at a hospital with greater occupational stress

Item 8. I prefer a subspecialty that will provide a high salary

Item 9. I prefer a subspecialty with prestigious experts

Item 10. I prefer a subspecialty with good career prospects

Item 11. I prefer a more competitive subspecialty

Item 12. I prefer an interesting subspecialty

Item 13. I prefer a subspecialty with greater job satisfaction

Item 14. I prefer a subspecialty that fits my character and work style

Item 15. I prefer a subspecialty that will have a limited effect on my leisure time

Item 16. I prefer a subspecialty with few night or overtime shifts

Item 17. I am willing to choose a subspecialty where I will always be on-call

Item 18. I am willing to choose a subspecialty that is recommended by family or friends

Item 19. I am willing to choose a subspecialty where I can serve my relatives

Item 20. I am willing to choose a subspecialty with a greater likelihood of patient-physician conflict
