# Supplementary material for: Development of a career questionnaire for medical undergraduates using Mokken scale analysis
Source: BMC Med Educ. 2022 Apr 15;22:286. doi: 10.1186/s12909-022-03340-8 (PMC9011374; doi:10.1186/s12909-022-03340-8)
Supplement: Supplementary file 3 — Additional file 3: Supplementary Fig. 1. Items score plot. Boxplots represent the fraction of different scores for each item. [file 12909_2022_3340_MOESM3_ESM.docx]

Additional files 3.

Supplementary Figure 1 Items score plot


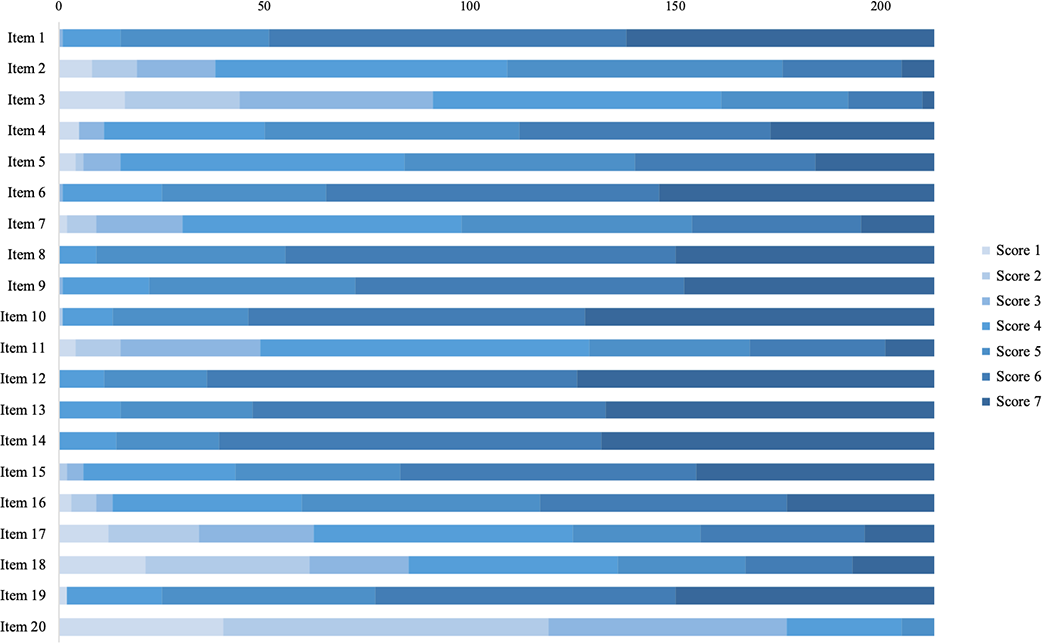


Boxplots represent the fraction of different scores for each item, range from 1 to 7(light to dark). Item 1. I prefer to choose general and famous hospital（i.e. tertiary hospitals）

Item 2. I prefer to choose medium degree hospital（i.e. secondary hospitals）

Item 3. I prefer to meet the needs of society (i.e., community or private hospitals) Item 4. I prefer to work at a specialized hospital with a good reputation

Item 5. I prefer to work at a hospital with room for promotion Item 6. I prefer to work at a hospital near my hometown

Item 7. I am willing to work at a hospital with greater occupational stress Item 8. I prefer a subspecialty that will provide a high salary

Item 9. I prefer a subspecialty with prestigious experts Item 10. I prefer a subspecialty with good career prospects Item 11. I prefer a more competitive subspecialty

Item 12. I prefer an interesting subspecialty

Item 13. I prefer a subspecialty with greater job satisfaction

Item 14. I prefer a subspecialty that fits my character and work style

Item 15. I prefer a subspecialty that will have a limited effect on my leisure time Item 16. I prefer a subspecialty with few night or overtime shifts

Item 17. I am willing to choose a subspecialty where I will always be on-call

Item 18. I am willing to choose a subspecialty that is recommended by family or friends Item 19. I am willing to choose a subspecialty where I can serve my relatives

Item 20. I am willing to choose a subspecialty with a greater likelihood of patient-physician conflict
