# Supplementary material for: Development of a career questionnaire for medical undergraduates using Mokken scale analysis
Source: BMC Med Educ. 2022 Apr 15;22:286. doi: 10.1186/s12909-022-03340-8 (PMC9011374; doi:10.1186/s12909-022-03340-8)
Supplement: Supplementary file 4 — Additional file 4: Supplementary Fig. 2. Graphic display of monotonicity. One graph for each item plots the estimated item response function. [file 12909_2022_3340_MOESM4_ESM.docx]

Additional files 4.

Supplementary Figure 2 Graphic display of monotonicity. One graph for each item plots the estimated item response function.


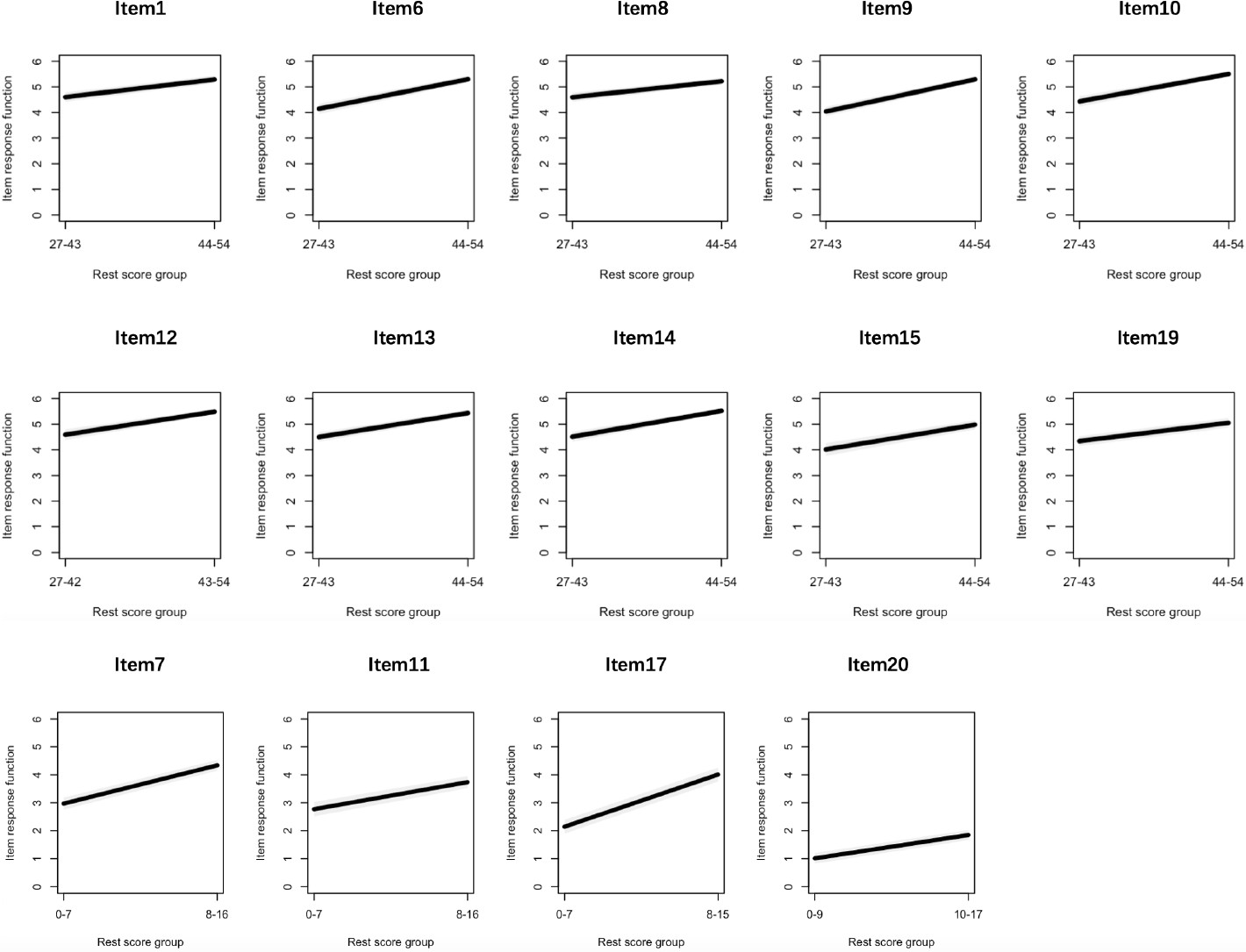


Item 1. I prefer to choose general and famous hospital（i.e. tertiary hospitals） Item 6. I prefer to work at a hospital near my hometown

Item 8. I prefer a subspecialty that will provide a high salary Item 9. I prefer a subspecialty with prestigious experts

Item 10. I prefer a subspecialty with good career prospects Item 12. I prefer an interesting subspecialty

Item 13. I prefer a subspecialty with greater job satisfaction

Item 14. I prefer a subspecialty that fits my character and work style

Item 15. I prefer a subspecialty that will have a limited effect on my leisure time Item 19. I am willing to choose a subspecialty where I can serve my relatives Item 7. I am willing to work at a hospital with greater occupational stress

Item 11. I prefer a more competitive subspecialty

Item 17. I am willing to choose a subspecialty where I will always be on-call

Item 20. I am willing to choose a subspecialty with a greater likelihood of patient-physician conflict
